# King George's Psychosis: A Case Report of Neuroporphyria in an Acute Psychiatric Inpatient Unit

**DOI:** 10.7759/cureus.115151

**Published:** 2026-08-25

**Authors:** Bruno Vidal, João Azenha, Daniela Jeremias, Joaquim Gago, Leonor Brito-Santana

**Affiliations:** 1 Psychiatry Department, Hospital de Egas Moniz, Lisbon, PRT

**Keywords:** acute porphyria, neuroporphyria, neuropsychiatric symptoms, porphyrinogenic drugs, psychosis

## Abstract

Porphyrias are rare metabolic disorders stemming from impaired heme biosynthesis, with neuroporphyrias comprising a subset characterized by neuropsychiatric symptoms due to toxic intermediary buildup in the nervous system.

Here, we explore the case of a 67-year-old man with no previous psychiatric history who was taken to the emergency department for psychiatric symptoms, including delusional speech, grandiosity, irritable mood, and psychomotor agitation. He was involuntarily admitted under the Portuguese Mental Health Act with a provisional diagnosis of mania with psychotic symptoms. Despite initial treatment with valproic acid, clinical deterioration prompted a metabolic workup leading to a diagnosis of acute porphyria. The patient was discharged fully recovered and remained stable during follow-up, relieved after understanding his diagnosis.

Although rare, porphyric attacks can be fatal or cause long-term neurological damage. This case underscores the importance of recognizing neuroporphyria, highlighting the need for comprehensive evaluation and timely intervention to mitigate potential adverse outcomes.

## Introduction

Porphyrias are a group of rare disorders of heme biosynthesis that can manifest as hepatic, neuropsychiatric, or cutaneous syndromes. These syndromes result from enzymatic defects in the heme biosynthetic pathway [1].

Neuroporphyrias (NP) manifest with neuropsychiatric symptoms due to toxic intermediate accumulation in the nervous system [1]. Four types are recognized, with common symptoms despite different heme synthesis impairments [1]. Acute intermittent porphyria (AIP), the most frequent type, affects 1 in 20,000 Caucasian individuals of Western European descent and usually presents with abdominal neuropathic pain, autonomic changes, and psychiatric symptoms [1,2], which can be the sole manifestation [2,3]. Sudden death from arrhythmias can occur.

NP are more common in individuals with mental illness and are often underdiagnosed due to clinicians' unfamiliarity [4,5]. The historical case of King George III's mental health decline, depicted in the movie "The Madness of King George," has been subject to interpretation, with some scholars proposing porphyria as the cause of his psychiatric symptoms [6,7]. This case report highlights diagnostic challenges and the importance of clinical suspicion to reduce morbidity and mortality.

## Case presentation

A 67-year-old man with no prior psychiatric history presented with progressive behavioral changes. Over two months, these escalated to marked irritability, grandiose speech, and delusions. The patient claimed connections to high-profile figures, such as the Queen of England, and complained of a facial rash. He was taken to the emergency department for urgent psychiatric evaluation, where he was severely agitated, uncooperative, and dysphoric and presented with flight of ideas and grandiose delusions. He refused treatment and was involuntarily admitted under the Portuguese Mental Health Act [8], with a provisional diagnosis of mania with psychotic symptoms, and treated with valproic acid and risperidone.

**Figure 1:**
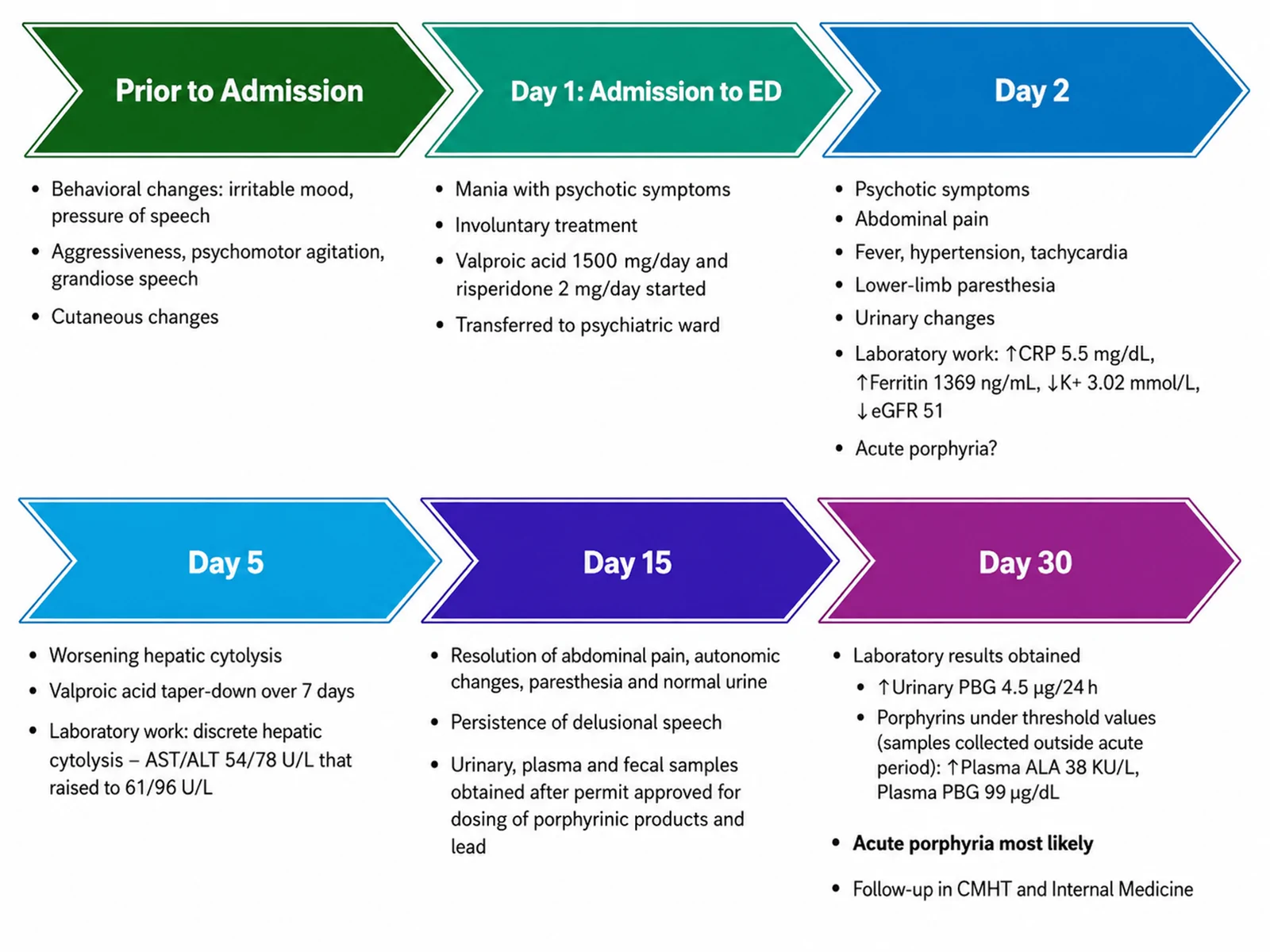
Timeline of neuropsychiatric symptoms, changes in clinical presentation, clinical evolution, and laboratory findings CRP: C-reactive protein; K+: potassium levels; eGFR: estimated glomerular filtration rate; AST: aspartate aminotransferase; ALT: alanine transaminase; PBG: porphobilinogen; ALA: delta-aminolevulinic acid; CMHT: Community Mental Health Team.

Soon after admission, he developed sudden abdominal pain, fever, hypertension, tachycardia, lower-limb paresthesia, and reddish-brown to dark-green urine, prompting further tests (see Figure 1 for timeline). Blood tests showed raised inflammatory markers, hypokalemia, and hepatic cytolysis.

After urgent observation by Internal Medicine, the hypothesis of NP was raised as most likely, and hemochromatosis, lead poisoning, and Wilson's disease were considered. Brain imaging and additional blood tests revealed no significant changes. A special permit was requested for measuring porphyrin precursors and lead levels, as these tests are not routinely available in our hospital. Brief cognitive assessment excluded cognitive impairment.

Valproic acid, a known porphyrinogenic drug, was gradually discontinued. Although the delusions initially persisted, the patient’s physical symptoms began to improve. On day 15 after admission, urinary and serum tests were performed, by which time the acute phase had already subsided. Porphobilinogen levels were elevated, supporting the diagnosis of acute NP. Urinary and fecal porphyrins, as well as δ-aminolevulinic acid (ALA) and porphobilinogen (PBG) serum levels, remained below the established thresholds; however, as previously noted, these samples were obtained outside the acute episode. Lead poisoning was excluded. Given the persistence of delusional symptoms, risperidone was replaced with oral paliperidone because of its more favorable safety profile in patients with porphyria [9]. Following further clinical improvement, the patient continued to show limited insight into his condition; therefore, to facilitate treatment adherence, oral paliperidone was transitioned to long-acting injectable paliperidone palmitate. After completion of the recommended initiation regimen, a maintenance dose of 100 mg IM monthly was established. The patient progressively achieved full remission of psychotic symptoms and was discharged after 48 days, with follow-up arranged in both psychiatry and internal medicine.

At subsequent outpatient assessments, he remained clinically well and expressed surprise at the diagnosis, while also reporting gratitude for the care received and his recovery. During the following two years, he remained free of psychotic symptoms, allowing for the gradual tapering and eventual discontinuation of antipsychotic treatment. No recurrence of psychiatric symptoms has since been observed, and he currently remains under follow-up exclusively by internal medicine, with no apparent complications reported. Written informed consent for publication of this case report was obtained from the patient.

## Discussion

New-onset psychosis requires evaluating psychiatric and organic causes. In this case, primary psychiatric conditions were unlikely, given the patient’s age and sudden appearance of abdominal pain, neuropathic symptoms, fever, and urinary changes. Investigation of secondary causes of neuropsychiatric symptoms like lead poisoning, hemochromatosis, Wilson's disease, and acute NP was key [10]. Given the pleomorphic presentation of NPs, diagnosing porphyria-induced psychosis is usually quite challenging. To reach an accurate diagnosis, a detailed anamnesis, physical examination, and complementary investigation comprising laboratory and neuroimaging examinations were performed in the psychiatry ward. Clinicians often rule out acute porphyria when there is no family history, considering the numerous potential causes of neurological symptoms and abdominal pain. However, given the low disease penetrance, family history may be absent, as symptomatic disease can skip generations [5,11].

The usual screening tests for NP include random urine PBG and ALA samples. Unfortunately, similar to our case, few hospital facilities have this rapid qualitative test, and a send-out test is the only way of assessment. This led to a significant delay in clinical management, but once the diagnosis became a possibility, appropriate treatment changes were put in place, allowing mitigation of the symptoms exacerbated by valproic acid [12].

Valproic acid is a known porphyrinogenic drug and can exacerbate porphyric attacks. Other porphyrinogenic psychotropics include barbiturates, carbamazepine, and tricyclic antidepressants [9].

Abdominal pain can occur in numerous clinical settings. The high ferritin values demanded assessment of possible causes of iron overload [10,13]. Iron kinetics, abdominal ultrasound, and brain MRI were performed and did not show significant changes. Wilson’s disease and hemochromatosis could be successfully excluded. Neuroimaging is typically normal in NP, though in rare cases, white matter changes or cortical atrophy may occur.

Considering the possible causes of neuropathies, lead poisoning was essential to exclude, given that it can cause neuropathy, psychiatric symptoms, and elevations in urinary porphyrins [10]. Lead poisoning does not, however, cause elevated urinary PBG, and lead values were negative, allowing us to safely exclude this clinical hypothesis [10].

In this scenario, the abdominal pain appeared after valproic acid was started and was concurrent with urinary changes, liver changes, neuropathy, and psychiatric symptoms (Figure 1). However, besides acute porphyrias, there are no other causes of abdominal pain that cause elevations of urinary PBG. The elevated urinary PBG, even when tested outside the acute window, strongly supported a diagnosis of acute NP, especially in the absence of other causes [11].

To distinguish between the NPs, laboratory work performed during the acute phase of symptoms is key [14]. Besides measurements of PBG and ALA in serum, urine, and fecal samples, porphyrinic products can also provide valuable information [15]. Genetic testing is advised for confirmation [14].

In summary, this case required a tailored approach, deviating from standard protocols in a psychiatry ward. The awareness of subtle clinical signs played a pivotal role in distinguishing and diagnosing the condition effectively.

## Conclusions

Although acute porphyrias are rare, they occur more frequently in psychiatric settings than in the general population. Effectively diagnosing and managing porphyria in a psychiatric setting is a challenge. Clinicians should maintain a high index of suspicion for porphyria in new-onset psychosis with atypical features. Misdiagnosis may lead to potentially hazardous use of porphyrinogenic drugs, such as valproic acid.

## References

[1] Jameson JL, Fauci AS, Kasper DL, Hauser SL, Longo DL, Loscalzo J (2018). Harrison’s Principles of Internal Medicine, 20th Edition. Harrison’s Principles of Internal Medicine. 20th ed.

[2] Altintoprak AE, Ersel M, Bayrakci A (2009). An unusual suicide attempt: a case with psychosis during an acute porphyric attack. Eur J Emerg Med.

[3] Ellencweig N, Schoenfeld N, Zemishlany Z (2006). Acute intermittent porphyria: psychosis as the only clinical manifestation. Isr J Psychiatry Relat Sci.

[4] Jara-Prado A, Yescas P, Sánchez FJ, Ríos C, Garnica R, Alonso E (2000). Prevalence of acute intermittent porphyria in a Mexican psychiatric population. Arch Med Res.

[5] Suarez JI, Cohen ML, Larkin J, Kernich CA, Hricik DE, Daroff RB (1997). Acute intermittent porphyria: clinicopathologic correlation. Report of a case and review of the literature. Neurology.

[6] Macalpine I, Hunter R (1966). The "insanity" of King George III: a classic case of porphyria. Br Med J.

[7] Macalpine I, Hunter R, Rimington C (1968). Porphyria in the royal houses of Stuart, Hanover, and Prussia. A follow-up study of George 3d's illness. Br Med J.

[8] Assembleia da República. Lei n.º 36/98. Lei de Saúde Mental (1998). Assembleia da República. Lei n.º 36/98: Lei de Saúde Mental. Diário da República Eletrónico. Diário da República Eletrónico.

[9] European Porphyria Network (ND). European Porphyria Network. Drug safety in porphyria. http://www.drugs-porphyria.org.

[10] Sood GK, Anderson KE (2018). Acute intermittent porphyria: pathogenesis, clinical features, and diagnosis. UpToDate.

[11] Elder G, Harper P, Badminton M, Sandberg S, Deybach JC (2013). The incidence of inherited porphyrias in Europe. J Inherit Metab Dis.

[12] Holroyd S, Seward RL (1999). Psychotropic drugs in acute intermittent porphyria. Clin Pharmacol Ther.

[13] Moghe A, Seetharam A, Garret C (2023). Elevated serum ferritin levels in acute intermittent porphyria may reflect time-related effects of hemin rather than iron overload. ResearchGate [PREPRINT].

[14] Gerischer LM, Scheibe F, Nümann A, Köhnlein M, Stölzel U, Meisel A (2021). Acute porphyrias - a neurological perspective. Brain Behav.

[15] Suh Y, Gandhi J, Seyam O, Jiang W, Joshi G, Smith NL, Ali Khan S (2019). Neurological and neuropsychiatric manifestations of porphyria. Int J Neurosci.

